# Vaccinated Toddler With 15B/15C Serotype Switch Pneumococcal Meningitis

**DOI:** 10.7759/cureus.90481

**Published:** 2025-08-19

**Authors:** Richard Kevorkian, Milene T Williams, Cory M Mcfadden, Brendan T Jones, Jason R Smedberg

**Affiliations:** 1 Infectious Disease Lab, Landstuhl Regional Medical Center, Landstuhl, DEU; 2 Microbiology, Landstuhl Regional Medical Center, Landstuhl, DEU; 3 Pediatrics, Landstuhl Regional Medical Center, Landstuhl, DEU; 4 Multidrug-Resistant Organism Repository and Surveillance Network (MSRN), Walter Reed Army Institute of Research, Silver Spring, USA

**Keywords:** invasive pneumococcal disease, meningitis, pcv13, pcv20, pediatric vaccine, streptococcus pneumoniae, vaccine coverage

## Abstract

*Streptococcus pneumoniae *(*S. pneumoniae*) remains a leading cause of bacterial pneumonia and meningitis in children globally, despite pneumococcal conjugate vaccine (PCV) implementation. We report a case of fatal pneumococcal meningitis in a fully vaccinated two-year-old male patient. Despite prior PCV13 vaccination, the patient developed meningitis caused by *S. pneumoniae *serotype 15B in the cerebrospinal fluid (CSF) and 15C in the blood. Genomic sequencing revealed nearly identical isolates differing by a capsular switch mutation. This case highlights the limitations of PCV13 in the context of emerging serotypes like 15B and 15C. The recent approval of PCV20, which includes serotype 15B, offers broader serotype coverage compared to PCV13. Based on these findings, we suggest that further research is warranted to investigate the potential role of PCV20, either as a booster for children under five previously vaccinated with PCV13 or in primary immunization schedules, to enhance protection against the evolving landscape of pneumococcal serotypes and reduce invasive disease. Continued surveillance and adaptive vaccination strategies are crucial, as *S. pneumoniae* continues to pose a significant threat to young children worldwide.

## Introduction

*Streptococcus pneumoniae* (*S. pneumoniae*) remains the leading cause of bacterial pneumonia and meningitis in children globally [1,2], contributing substantially to childhood morbidity and mortality. Its clinical manifestations encompass a range of pneumococcal diseases, including otitis media, pneumonia, and meningitis. *S. pneumoniae*’s prominence as a cause of bacterial meningitis in young children stems from its ability to exploit immature immunity and employ virulence factors like the polysaccharide capsule [3,4]. Its pathogenesis, from colonization to blood-brain barrier breach, underscores the importance of early intervention [1]. Pneumococcal vaccination is a crucial tool in preventing pediatric bacterial meningitis, a serious and potentially life-threatening infection caused by *S. pneumoniae*. The introduction of pneumococcal conjugate vaccines (PCVs) has significantly reduced the incidence of invasive pneumococcal disease (IPD), including bacterial meningitis, in children [5,6]. However, despite the success of PCVs, pneumococcal disease remains a public health concern due to factors such as incomplete vaccine coverage and the emergence of nonvaccine serotypes. This case report describes a fatal case of pneumococcal meningitis in a fully vaccinated child, highlighting the limitations of current PCV13 coverage in the face of serotype replacement. We discuss the clinical presentation, diagnosis, and management of this case, focusing on the implications for vaccine strategies and the importance of ongoing surveillance.

## Case presentation

Approximately two weeks prior to presentation, the patient's parents and sibling experienced a suspected viral gastroenteritis. Around the same time, the patient (a previously healthy two-year-old boy) developed intermittent fever, vomiting, and malaise. On day 2 of symptoms, he was evaluated in the ED and diagnosed with suspected viral gastroenteritis, receiving treatment with antiemetic medication. He was seen again in the ED on day 7 of symptoms, at which time laboratory tests revealed normal blood counts and serum electrolytes. He was presumptively diagnosed with a bacterial gastrointestinal infection and started on oral cefixime. This was discontinued two days later (day 9) at a follow-up ED visit due to the symptoms believed to be more consistent with a viral illness. His symptoms continued and progressed to include altered mental status over the next four days (days 9-13).

Subsequently, he presented to the ED on day 14 of symptoms, and the patient was noted to be febrile to 40°C, tachycardic, with altered mental status (responsive only to painful stimuli). Physical examination revealed neck rigidity, extensor posturing, and bilateral eye deviation suggestive of seizure activity, which was treated with benzodiazepines. Suspecting meningitis, a lumbar puncture was obtained. The cerebrospinal fluid (CSF) was cloudy, with analysis showing an elevated WBC count, neutrophils; glucose was critically low; protein was elevated; and the Gram stain was positive for numerous Gram-positive cocci in pairs and chains (Table 1). Blood work at the time showed no leukocytosis, hypokalemia (3.0 mmol/L), and elevated lactic acid (4.5 mmol/L). CSF and blood cultures were positive for* S. pneumoniae* with susceptibility to all tested antibiotics. He was started on IV antibiotics, and due to poor responsiveness, he was intubated and underwent a brain MRI for further evaluation.

**Table 1:** Cerebrospinal fluid and peripheral blood laboratory results from microscopic analysis and automated blood chemistry analyzers

	Laboratory value	Patient result	Normal range (2 years old)
Cerebrospinal fluid (CSF)	White blood cell count (WBC)	2,500 cells/mm^3^	<5-10 cells/mm^3^
	Gram stain	Positive for Gram-positive cocci in pairs and chains	N/A
	Neutrophils (%)	81%	0-70%
	Lymphocytes (%)	15%	30-75%
	Monocytes (%)	4%	0-10%
	Glucose (mg/dL)	10 mg/dL	50-80 mg/dL
	Protein (mg/dL)	200 mg/dL	15-45 mg/dL
Blood work	Gram stain	Positive for Gram-positive cocci in pairs and chains	N/A
	White blood cell count (WBC)	10,800 cells/mm^3^	9-14,000 cells/mm^3^
	Potassium (mmol/L)	3.0 mmol/L	3.8-5.2 mmol/L
	Lactic acid (mmol/L)	4.5 mmol/L	0.7-2.5 mmol/L

Due to clinical instability, the patient was transferred to a local facility with a pediatric intensive care unit. Despite aggressive treatment, he remained comatose with minimal response. Echocardiography at that time showed a new vegetation on the mitral valve. Over the course of a few days, he developed dramatic lability in his vital signs, and an emergent MRI showed impending brainstem herniation. A brain death exam was performed, confirming brain death, and care was withdrawn in order to pursue organ donation.

Two isolates were recovered, one from the patient’s blood and the other from the patient’s CSF. Both isolates were sequenced and genomes annotated (accession no. SAMN47824484 and SAMN47824485). Both isolates belong to sequence type 199. The only antimicrobial resistance genes carried by the two isolates were the macrolide resistance genes mef(A) and msr(D). However, the CSF isolate belonged to serotype 15B while the blood isolate belonged to serotype 15C.

## Discussion

This case of *S. pneumoniae *meningitis in a fully vaccinated two-year-old boy underscores the ongoing threat of IPD in young children despite widespread vaccination. The two *S. pneumoniae *isolates were nearly identical when sequenced from the patient’s blood and CSF, differing by just four single-nucleotide polymorphisms and an INDEL (INsertion or DELetion of nucleotides). Despite their similarity, they belonged to two different serotypes: 15B in the CSF and 15C in the blood. This divergence was due to a two-nucleotide duplication (c.428_429dup) in the wciZ gene of the blood isolate, causing a frameshift mutation and a truncated WciZ O-acetyltransferase protein. This inactivation prevents acetylation in the capsular pentasaccharide repeating unit, driving a switch from serotype 15B to 15C [7]. This capsular switch is notable because it can occur at a high frequency in some isolates, though it may not be reversible in others [8,9]. Since only one isolate was sequenced per site, it remains unclear whether both serotypes coexisted at both locations.

The presence of serotypes 15B and 15C in this case highlights the phenomenon of serotype replacement, a consequence of prolonged use of PCVs like PCV13. The introduction of PCVs has significantly reduced the incidence of IPD caused by vaccine-targeted serotypes [5]. However, this success has been offset by the emergence of nonvaccine serotypes, such as 15B and 15C, which have increasingly driven IPD cases [10]. In this instance, the patient had received the full schedule of PCV13, which offered no protection against serotypes 15B and 15C, revealing a gap in coverage that allowed this severe infection to develop [11]. A limitation of our analysis is that only one isolate per site (blood and CSF) was sequenced. This limits our certainty regarding the full distribution of serotypes within each compartment. It is possible that other serotypes were present but not detected due to this sampling bias. Future studies should consider sequencing multiple isolates from each site to provide a more comprehensive understanding of serotype dynamics in IPD. Additionally, the capsular switch may have occurred in vivo in response to immune pressure.

Serotype 15B is included in PCV20 (approved for infants in April 2023). While serotype 15C is not covered by PCV20, its limited immune evasion suggests likely cross-protection [11,12]. In contrast, PCV7, PCV13, and PCV15 do not protect against either serotype 15B or 15C (Table 2) [11]. The patient’s progression to meningitis despite the PCV13 vaccination illustrates the urgent need for updated management approaches to address these emerging serotypes.

**Table 2:** Streptococcus pneumoniae serotypes included in pneumococcal conjugate vaccines (PCV7, PCV10, PCV13, PCV15, and PCV20) and their respective public availability dates for children under five PCV: pneumococcal conjugate vaccine

Serotype	PCV7 (2000)	PCV10 (Not U.S., 2009)	PCV13 (2010)	PCV15 (2022)	PCV20 (pediatric 2023)
4	✓	✓	✓	✓	✓
6B	✓	✓	✓	✓	✓
9V	✓	✓	✓	✓	✓
14	✓	✓	✓	✓	✓
18C	✓	✓	✓	✓	✓
19F	✓	✓	✓	✓	✓
23F	✓	✓	✓	✓	✓
1	-	✓	✓	✓	✓
5	-	✓	✓	✓	✓
7F	-	✓	✓	✓	✓
3	-	-	✓	✓	✓
6A	-	-	✓	✓	✓
19A	-	-	✓	✓	✓
22F	-	-	-	✓	✓
33F	-	-	-	✓	✓
8	-	-	-	-	✓
10A	-	-	-	-	✓
11A	-	-	-	-	✓
12F	-	-	-	-	✓
15B	-	-	-	-	✓
15A	-	-	-	-	-
15C	-	-	-	-	-

PCV20 represents a significant advancement in pneumococcal disease prevention. PCV13 was the standard until PCV20 licensure in 2023. While PCV15 was licensed in 2022, its adoption has been limited in some regions due to its relatively modest increase in serotype coverage compared to PCV13 [13,14]. PCV20 offers enhanced protection by including additional serotypes, such as 15B, that are absent from PCV13 [6]. This broader coverage is critical as non-PCV13 serotypes (22F, 33F, 8, 10A, 11A, 12F, and 15B) have become prominent drivers of IPD following PCV13’s widespread use [10]. By addressing serotype replacement, PCV20 provides a more robust defense against pneumococcal disease, including meningitis, until new nonvaccine serotypes emerge.

When given as a single dose to very young children fully vaccinated with PCV13, the PCV20 elicited immune responses expected to provide protection against the seven additional serotypes included in PCV20, supporting the use of a PCV20 booster shot to children under five who were previously vaccinated with PCV13 [15]. Based on this case, further investigation into the feasibility and potential benefits of administering a PCV20 booster shot to children under five who have previously received PCV13 may be warranted. To the author's knowledge, there are no published cost-effectiveness analyses of PCV20 in pediatric populations. Such analyses are crucial to inform evidence-based decisions regarding the potential implementation of PCV20 in routine immunization schedules. Our suggestion to consider PCV20 inclusion in immunization schedules is based primarily on the broader serotype coverage offered by this vaccine and the promising immunogenicity data from clinical trials. However, we acknowledge the absence of robust pediatric outcome data demonstrating a direct reduction in IPD following widespread PCV20 implementation. Prospective surveillance studies are needed to assess the real-world impact of PCV20 on pediatric IPD rates and to confirm its effectiveness in preventing disease caused by emerging serotypes.

Differences in vaccine schedules between the USA and Europe, and time needed to observe disease declines, shape *S. pneumoniae *epidemiology [5,6,16]. This variability underscores the importance of ongoing surveillance and adaptive vaccination strategies to sustain PCV effectiveness. Vaccination, particularly with PCV20, represents a cornerstone of prevention, reducing both colonization and invasive disease [14,17,18]. This is vital for children under five, where non-PCV13 serotypes increasingly cause IPD [19].

## Conclusions

In conclusion, this case underscores the continued importance of *S. pneumoniae* as a cause of morbidity and mortality in children, even in the context of existing PCV13 vaccination programs. The observed serotype switch from 15B to 15C highlights the need for ongoing surveillance of pneumococcal serotypes and further investigation into the evolving landscape of pneumococcal disease. Specifically, future research should focus on understanding the prevalence of non-PCV13 serotypes, assessing the potential for cross-protection, and evaluating the feasibility and cost-effectiveness of incorporating PCV20, either as a booster dose or as part of the primary immunization schedule. This case serves as a reminder of the dynamic nature of pneumococcal disease and the importance of adaptive vaccination strategies.
